# The association between Hba1c and arterial stiffness among non-diabetic patients with chronic kidney disease

**DOI:** 10.1590/1677-5449.200245

**Published:** 2021-06-16

**Authors:** Atakan Turgutkaya, Gülay Aşçı

**Affiliations:** 1 Adnan Menderes University – ADÜ, Hematology Department, Aydın, Turkey.; 2 Ege University – EÜTF, Ege University Hospital, Bornova, İzmir, Turkey.

**Keywords:** arterial stiffness, atherosclerosis, cardiovascular, chronic kidney disease, pulse wave velocity, rigidez arterial, aterosclerose, cardiovascular, doença renal crônica, velocidade da onda de pulso

## Abstract

**Background:**

Cardiovascular events are seen more frequently after the age of 60 and they are a significant cause of morbidity and mortality. Arterial stiffness is a property that can be expressed by pulse wave velocity and this value is assumed to be a predictor of cardiovascular events. Patients with chronic kidney disease and dysregulated blood sugar have increased atherosclerosis and arterial stiffness, but the relationship between physiological levels of Hba1c and arterial stiffness is less clear in chronic kidney disease patients without diabetes mellitus.

**Objectives:**

Here, we aimed to investigate the degree of arterial stiffness among non-diabetic, non-dialysis dependent chronic kidney disease patients with physiological HbA1c levels.

**Methods:**

We enrolled 51 patients who were followed up at Ege University Hospital Nephrology Department between February and June 2015. Non-diabetic, non-dialysis dependent chronic kidney disease patients were included in the study. Blood pressure and pulse wave velocity were measured with an applanation tonometry device (Sphygmocor Vx Software Atcor Medical, Australia). Correlations between pulse wave velocity and the aforementioned parameters were investigated (see below).

**Results:**

We detected a significant correlation between pulse wave velocity and systolic blood pressure (p=0.0001) and Hba1c (p=0.044) separately. There was an inverse correlation with creatinine clearance (p=0.04). We also detected a significant correlation with serum phosphorus level (p=0.0077) and furosemide use (p=0.014). No correlations were found among the other parameters.

**Conclusions:**

Arterial stiffness is an important predictor of cardiovascular events and measuring it is an inexpensive method for estimating morbidity and mortality. Our study supports the importance of measuring arterial stiffness and of controlling blood glucose levels, even at physiological Hba1c values, especially for chronic kidney disease patients.

## Resumo

**Contexto:**

Os eventos cardiovasculares são mais frequentes em pessoas com mais de 60 anos, e sugere-se que sejam causas significativas de morbidade e mortalidade. A rigidez arterial é expressa pela velocidade da onda de pulso, e presume-se que esse valor seja um preditor de eventos cardiovasculares. Os pacientes com doença renal crônica e desregulação do açúcar no sangue têm aterosclerose e rigidez arterial aumentadas. No entanto, a relação entre os níveis fisiológicos de Hba1c e a rigidez arterial entre pacientes com doença renal crônica sem diabetes melito é menos clara.

**Objetivos:**

Buscamos investigar o grau de rigidez entre os pacientes sem diabetes e portadores de doença renal crônica sem dependência de diálise com níveis fisiológicos de HbA1c.

**Métodos:**

Incluímos 51 pacientes, sem diabetes e com doença renal crônica sem dependência de diálise, que estavam em acompanhamento no Departamento de Nefrologia do Ege University Hospital entre fevereiro e junho de 2015. A pressão sanguínea e a velocidade da onda do pulso foram medidas com um dispositivo de tonometria de aplanação (Sphygmocor Vx Software Atcor Medical, Austrália). A correlação entre a velocidade da onda de pulso e os parâmetros mencionados anteriormente foi investigada.

**Resultados:**

Detectamos correlação significativa entre a velocidade da onda de pulso e a pressão arterial sistólica (p = 0,0001) e Hba1c (p = 0,044) separadamente. Foi constatada correlação inversa com a depuração de creatinina (p = 0,04). Também detectamos correlação significativa com o nível de fósforo sérico (p = 0,0077) e o uso de furosemida (p = 0,014). Não foi encontrada correlação entre outros parâmetros.

**Conclusões:**

A rigidez arterial é um preditor importante de eventos cardiovasculares, e a sua medição é um método de baixo custo para estimar a morbidade e mortalidade. Nosso estudo corrobora a importância da medição da rigidez arterial e do controle dos níveis de glicemia sanguínea mesmo em valores de Hba1c fisiológicos, principalmente para pacientes portadores de doença renal crônica.

## INTRODUCTION

Arterial stiffness (AS) refers to the rigidity of arterial vessels and is the result of a multifactorial process with extensive vascular calcifications in chronic kidney disease.^1^ It has prognostic value in kidney and cardiovascular system disorders and contributes to estimation of cardiovascular events (CVE).^2^^-^5 Progression to end-stage kidney disease increases AS (and vice versa), which is measured between the carotid and femoral arteries and expressed by pulse wave velocity (PWV).^1^ That atherosclerosis accelerates the effect of dysregulated blood sugar (expressed by a higher glycated hemoglobin [Hba1c] value) among chronic kidney disease (CKD) patients has been well demonstrated in clinical trials, but this is less clear among patients without diabetes.^4^^,^^6^ In our study, we investigated the relationship between AS and HbA1c values, other blood and urine parameters, smoking, and drug use in non-diabetic CKD patients.

## METHODS

Fifty-one CKD patients (31 males and 20 females) who were followed up at Ege University Hospital Nephrology Department between February and June 2015 were included in the study, which was designed to be single center, prospective, multidisciplinary, analytical, and cross-sectional. Being over the age of 18 years and having stage 2-5(non-dialysis) CKD were defined as the inclusion criteria; whereas the exclusion criteria were defined as having DM or stage 1 CKD, using anti-diabetic or immunosuppressive drugs, receiving renal replacement therapy, having active infection, malignity, or an immunosuppressive condition, and being pregnant or breastfeeding. Patients who were previously enrolled on any other clinical trial or unwilling to participate into the study were also excluded. Sample size analysis determined that 23 patients were required to achieve a power of 80% (standardized effect size: 0.82 with a 5% type I error margin).

The stage of CKD is determined by glomerular filtration rate (GFR).^7^ All patients were interviewed for habits of smoking or drug use. A Body Mass Index (BMI) calculation was performed for each of them. Patients’ blood pressure and PWV were measured with an applanation tonometry device (Sphygmocor Vx Software Atcor Medical, Sydney, Australia)., It was verified that patients were in a fasting state before measurement. Participants were instructed to avoid smoking and caffeine consumption before being measured. On the measurement day, blood and 24-hour urine samples were collected from the patients and sent to the biochemistry laboratory for blood count, Hba1c, urea, creatinine, uric acid, electrolytes (Na, K, Cl, Ca, Mg, P), albumin, globulin, fasting glucose, and parathormone assays (from blood) and for protein, electrolytes, and creatinine clearance tests (from urine). Measurements were performed in 1 session for each patient, using the carotid-femoral PWV measurement method with sequential applanation tonometry of the carotid and femoral regions, while ECG recording was also used to synchronize the R peak with the two different pulse waves in order to obtain transit time. Carotid-femoral distance was obtained by subtracting the distance from the carotid measurement site to the sternal notch from the distance from the sternal notch to the femoral measurement site. PWV was calculated by dividing the resulting distance by transit times between femoral and carotid points.^8^

### Ethical statement

All procedures were conducted in accordance with the ethical standards of the national research committee and with the 1964 Helsinki declaration and its later amendments or comparable ethical standards. Ethical committee approval was obtained for the study (14-9.1/11). Informed consent forms were obtained from all patients for participation and publication.

### Statistical analysis

Statistical assessment was performed using the SPSS 15.0 (SPSS Inc., Chicago, IL, USA) software program. Conformity of numerical variables to normal distribution was measured with the Shapiro Wilks test. Descriptive statistics for numerical variables were expressed as mean ± standard deviation (SD) and median (minimum-maximum), whereas counts and percentages were used for categorical data. Linear relationships between two numerical variables were examined by Pearson correlation analysis if parametric test assumptions were met or by Spearman correlation analysis if they were not. The results were evaluated within a 95% confidence interval and a p value below 0.05 was considered significant.

## RESULTS

**Figure 1:**
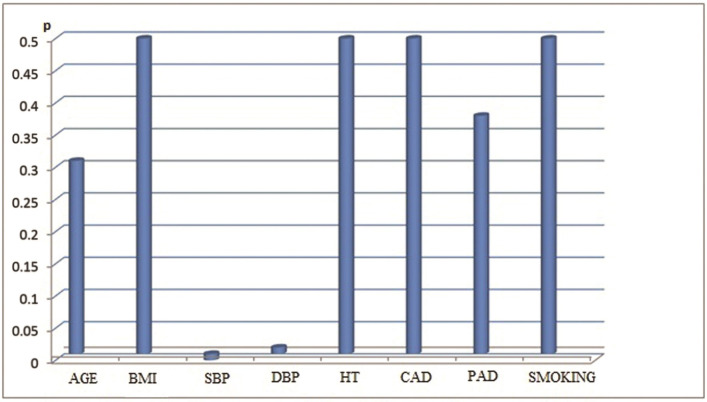
The Correlation between Pulse Wave Velocity (PWV) and Descriptive Parameters of the Patients.

**Figure 2:**
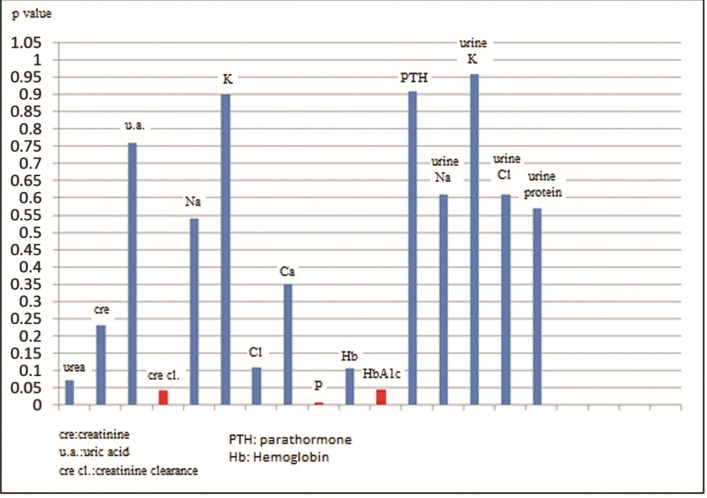
The Correlation between Pulse Wave Velocity (PWV) and Laboratory Parameters of the Patients.

**Figure 3:**
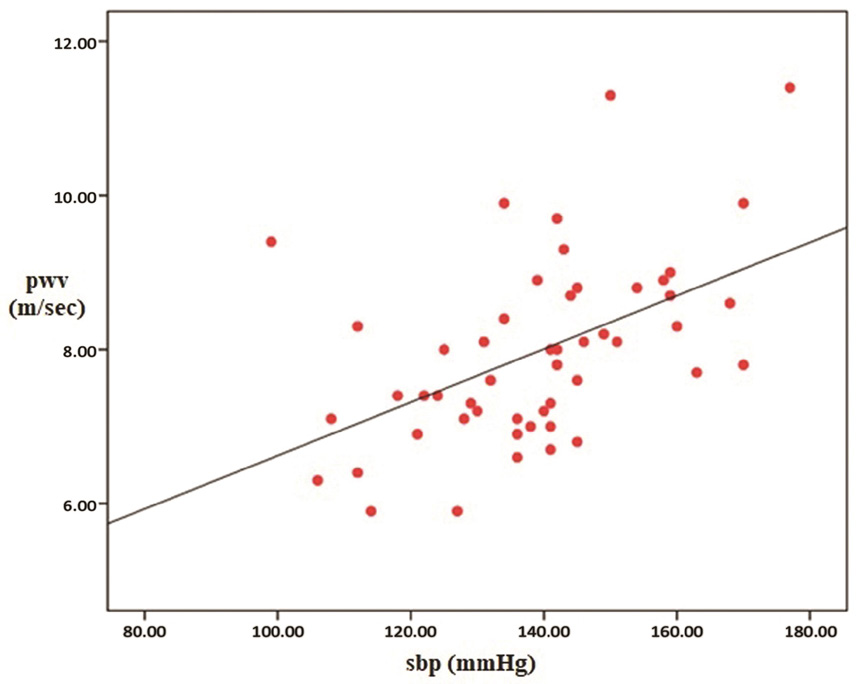
The Correlation between Pulse Wave Velocity (PWV) and Systolic Blood Pressure (SBP).

**Figure 4:**
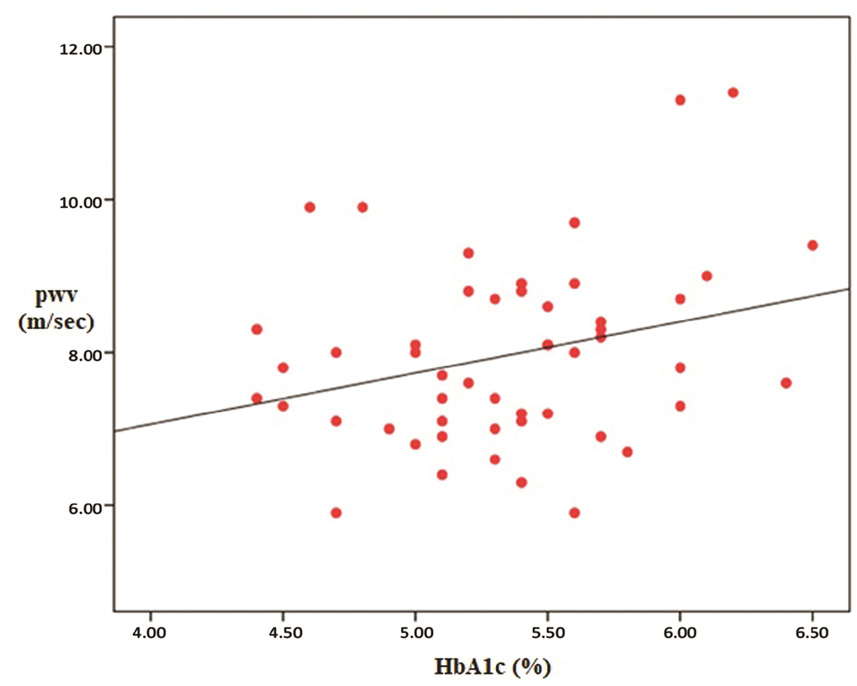
The Correlation between Pulse Wave Velocity (PWV) and Glycated hemoglobin (Hba1c).

Our study was performed with 51 patients (31 male/20 female), with non-diabetic, stage 2-5 non-dialysis dependent chronic kidney disease. The mean age of the patients was 57.4 ± 16.3 and the mean BMI value was 27. The mean systolic (SBP) and diastolic blood pressure (DBP) of the patients were 138 ± 17 mm Hg and 80 ± 15 mm Hg respectively. The mean HbA1c value of the cases was calculated as 5.3%. Seventeen percent of 51 patients had previously-known coronary arterial disease and 3% had peripheral arterial disease. When they were classified according to the causes of CKD; it was determined that 19.6% of the cases had glomerulonephritis, 15.6% had tubulointerstitial nephritis and drug-related causes, 17.6% had hypertension (HT), 13.7% had urolithiasis (and other obstructive causes), and 5.8% had polycystic kidney disease. The causative pathology could not be determined in 27.4% of the cases. Regarding drug use, 23% were taking aspirin, 5% were on antihyperlipidemic drugs, 33% were taking calcium channel blockers, 35% were on beta blockers, 33% were taking angiotensin-converting-enzyme (ACE) inhibitors, and 27% were receiving furosemide. Seventeen percent of the cases used to have a smoking habit at the time of enrollment on the study. The descriptive data and laboratory parameters of the cases are shown in Table 1 and Table 2, respectively. Regarding PWV values measured by the applanation tonometry device; the minimum, maximum and mean values were determined as 59 m/sec, 11.4 m/sec and 7.96 m/sec respectively (SD: 1.19). Parameters correlated with PWV were determined as HbA1c (p=0.044), creatinine clearance (p= 0.04), SBP (p= 0.0001), DBP (p= 0.02), and serum phosphorus value (p= 0.0077). Of these, creatinine clearance was inversely correlated with the PWV value. Additionally, a significant relationship was determined between the use of furosemide and PWV, but not between urine Na and PWV (p= 0.014 for furosemide and p= 0.6 for urine Na). When a multiple analysis method was used, it was detected that PWV was independently affected by only SBP and HbA1c. The correlations between PWV and the aforementioned parameters (descriptive parameters [Figure 1], laboratory parameters [Figure 2], SBP [Figure 3] and Hba1c [Figure 4]) are shown below.

**Table 1:** Descriptive Data of the Cases.

	Minimum	Maximum	Median (±SD)
Age	28	89	57.4±16.3
SBP (mm Hg)	99	177	138.7±17.3
DBP (mm Hg)	41	115	80.2±15.1
BMI (kg/m2)	20.7	41.3	27.06±4.2
CKD duration(months)	24	372	67.7±64.2
HbA1c(%)	4.4	6.4	5.3*

* Mean.

**Table 2:** Laboratory parameters of the patients.

	Minimum	Maximum	Median±SD
HbA1c %	4.4	6.5	5.3±0.5
Creatinine Clearance	9	64	31.5±16.1
Urea (serum) (mg/dl)	28	223	90.5±41.7
Creatinine (serum) (mg/dl)	0.77	5.8	2.5±1.1
Uric Acid (mg/dl)	3.6	13.7	7.2±1.8
Na (serum) (mEq/l)	128	146	139±3.8
K (serum) (mEq/l)	3.7	5.9	4.7±0.5
Cl (serum) (mEq/l)	89	111	102±5.3
Ca (serum) (mEq/l)	7.6	10.7	9.2±0.5
P (serum) (mEq/l)	1.7	5.8	3.77±0.8
Hb (g/dl)	7.4	16.4	12.8±1.78
PTH (pg/mL)	48.3	1026.8	261.4±218.3
Na (Urine) (mEq/24h)	24	277	126.06±53
K (Urine) (mEq/24h)	16	89	41.7±17.8
Cl (Urine) (mEq/24h)	21	245	101.5±51.3
Protein (Urine) (mg)	60	7680	1397±1633.2

Ca: Calcium; Cl: Chloride; h: hours; Hb: Hemoglobin; K: Potassium; Na: Sodium; P: Phosphorus; PTH: parathyroid hormone.

## DISCUSSION

Arterial stiffness is a property that is measured as the PWV between the carotid and femoral arteries, with the unit m/sec. Different causes contribute to AS, of which HT, DM, smoking, hypercholesterolemia, and aging are the most prominent.^9^ Obesity and salt consumption can be considered as other causes.^10^^,^^11^ Arterial stiffness is considered a prognostic marker for cardiovascular and kidney related morbidity and mortality.^5^ It has been suggested that AS is related to decreased GFR and accelerated progression to end stage kidney disease and it also leads to left ventricular hypertrophy and decreased coronary perfusion.^1^^,^^3^ Every 10 mL/min decrease in GFR has been shown to be associated with 0.4 m/sec increase in PWV.^12^ Our study supported the presence of an independent significant PWV relationship with both SBP and HbA1c level. The relatively lower frequency of HT in our patients (17.6%) was attributed to tight control of volume overload and blood pressure at our center. One interesting result was detection of no correlation between PWV and age, since increased arterial stiffness has been shown to be closely related with advanced age.^13^ This may be due to the limited patient number or to relatively lower PWV values in our study, which can be attributed to relatively younger population (32 patients [62.7%] younger than 60 and 12 patients [23.5%] over the age of 70 years). With regards to drug use, the correlation between PWV and furosemide (p= 0.014) disappeared when the multiple analysis method was used. This may be associated with the volume depleting and SBP depleting effect of furosemide, because increased volume contributes to AS by increasing arterial distension.^14^ However we did not detect any relationship between urine Na and PWV values. This can be explained by the reduced natriuresis capability as CKD progresses. There were no significant relationships with other antihypertensive agents such as beta blockers (p=0.27), calcium channel blockers (p=0.75), or ACE inhibitors (p=0.6). This finding was unexpected, especially for ACE inhibitors, because of their volume depleting effects and the reason therefore remains unknown. There was a correlation between PWV and serum phosphorus level (p=0.0077), but this disappeared when the multiple analysis method was used. There was no significant association between PWV and either serum calcium or parathyroid hormone (PTH) levels, even though increased PTH has been found to be linked with endothelial dysfunction and increased aortic pressure, chronic inflammation, vascular calcification, and atherosclerosis in some studies.^15^^-^17 Regarding the HbA1c-AS relationship, a study by Cavero-Redondo et al.^18^ suggested there was a positive correlation among 220 non-diabetic adult patients, similar to our study. Another study, by Zeng et al.,^19^ also supported the positive correlation between HbA1c and AS among 11014 patients and the finding maintained significance among 3553 individuals with normal glucose tolerance. In our study, age, sex, serum uric acid levels, smoking, antihyperlipidemic drug use, BMI, and CKD duration were not found to have significant correlations with PWV, conflicting with some studies.^20^^-^24 The main limitation of our study is that it has a relatively heterogeneous population due to the age range of 28-89 and GFR in the range of 9-64 mL/min, although it excluded stage 1 and dialysis-dependent patients.

## CONCLUSION

It is a well-known fact that AS, which is accepted as a predictor of CVE with morbidity and mortality, is expected to be increased in individuals with CKD ± insulin resistance or uncontrolled blood sugar regulation.^6^^,^^25^ To delay the progression of the microvascular complications of diabetes, including diabetic kidney disease; target HbA1c is recommended to be approximately 7%, except for patients at risk of hypoglycaemia.^26^ Our study detected an independent correlation between PWV and Hba1c, and between PWV and SBP. The most important result of our study is that the higher the HbA1c value (even at non-diabetic level), the higher the PWV and therefore the AS value in CKD patients. It is essential to focus on reduction of AS and hence the reduction of CVE by tightly controlling SBP and HbA1c levels in patients with CKD.
